# Do Cone Beam CT Picture Archiving and Communication Systems Viewer Interfaces Meet the Expectations of Dental Professionals From a Usability Perspective?

**DOI:** 10.7759/cureus.54288

**Published:** 2024-02-16

**Authors:** Yaren Dogan, Yigit Sirin

**Affiliations:** 1 Department of Oral and Maxillofacial Surgery, Istanbul University Institute of Health Sciences, Istanbul, TUR; 2 Department of Oral and Maxillofacial Surgery, Istanbul University Faculty of Dentistry, Istanbul, TUR

**Keywords:** cone beam computed tomography, user satisfaction, system usability scale, usability, picture archiving and communication system

## Abstract

Background

Cone beam computed tomography (CBCT) has revolutionized dental and maxillofacial imaging by providing high-resolution 3D visualizations, essential for accurate diagnosis and treatment planning. Despite its clinical advancements, the usability of CBCT viewer interfaces, which play a crucial role in the effective interpretation of imaging data, remains a critical concern.

Objective

This study aims to evaluate the usability of a CBCT viewer interface in clinical settings, focusing on the effectiveness, efficiency, and satisfaction perspectives, to identify potential areas for improvement.

Methods

Twenty-two participants (N=22) were assigned the task of locating the mental foramen in a mandible dataset, selected randomly, using the multiplanar reconstruction (MPR) mode of a CBCT viewer interface on a local network. The task's effectiveness was gauged by the completion rate, while efficiency was measured through the duration of the task, the number of mouse clicks, and the cursor's path in both pixels and meters. Satisfaction or perceived usability was evaluated using the system usability scale (SUS-TR), and computer system usability questionnaire (T-CSUQ), among other scales, with participants also providing open-ended feedback. Demographic characteristics served as classification variables.

Results

All participants completed the given task. No demography-related differences in efficiency were observed. Perceived usability (SUS-TR: 60.68±19.58, T-CSUQ: 43.63±16.34) was below the industry standards, categorizing the system as a detractor. Commonly reported issues included accessing the MPR window, cursor behavior, and unclear error messages. The mean SUS-TR score negatively correlated with efficiency-related variables (p<0.05 for each).

Conclusions

The study's findings indicate that the CBCT viewer interface does not fully meet dental professionals' usability expectations, as evidenced by the task's completion rate, efficiency metrics, and below-average usability scores. Despite the successful task completion by all participants, the identified issues in interface design, such as difficulties in accessing the MPR window and unclear error messages, highlight significant areas for improvement. To enhance user satisfaction and efficiency, future developments of CBCT viewer interfaces should incorporate feedback from end-users and prioritize user-friendly design principles.

## Introduction

Cone beam computed tomography (CBCT) is an X-ray-based imaging technology that has been widely utilized for over two decades. These devices address the limitations of conventional 2D imaging, such as distortion, magnification, and superimposition. Their practical advantages encompass a smaller footprint, lower radiation dose, and relative cost-effectiveness compared to medical tomography devices employed for similar purposes [1,2]. Although CBCT devices capture volumetric data, the computer screens used for image evaluation can only display 2D images. Consequently, the original volume must be stored and processed into 2D datasets, which can be examined from various planar orientations. Dental professionals assess the images on the screen using different display modes, with the most commonly employed modes being multiplanar reconstruction/reformatted (MPR), oblique coronal sections, and 3D reconstructions [2,3]. MPR enables the simultaneous and interactive visualization of the data from various planes and it proves invaluable for swiftly assessing spatial relationships.

Compared to 2D systems, CBCT data is considerably robust, necessitating a specific storage environment accessible and manipulable through dedicated software interfaces. Consequently, picture archiving and communication systems (PACS) are employed to securely store and digitally transmit electronic images. This is a sophisticated, fast-operating computer network system focused on the management of radiologic imagery - including ultrasound, X-ray, CT scans, PET scans, endoscopic procedures, and magnetic resonance imaging - which is at the forefront of medical imaging technology. The use of PACS eliminates the need for manual filing, storage, retrieval, and transmission of sensitive information. In terms of the storage, access, distribution, and presentation of images, PACS far surpasses traditional film archives [4]. With these systems, users can access the radiologist's reports, evaluate them alongside the patient's other findings, forward them to individuals or institutions outside the corporate network, and, when necessary, assess the entire dataset at resolutions tailored to their needs. The evaluation of cross-sectional data using a dedicated software interface involves numerous technical functions, demanding an adequate skill set and adaptation to the relevant software interface, independent of an individual's level of professional knowledge.

The interface design or optimization is an interdisciplinary domain within the field of human-computer interaction. It draws on insights from social and behavioral sciences, as well as computer science, and is commonly explored within the broader framework of user experience concept (UX) [5]. Traditional usability, a subset of UX, is defined by the International Organization for Standardization as "the extent to which a system, product, or service is employed by specified users to achieve predefined goals effectively, efficiently, and with satisfaction within a specific context" [6]. Effective product utilization necessitates users achieving accuracy and completeness for specific purposes. Efficiency is assessed by measuring the resources employed through various methods to achieve the accuracy and integrity of objectives. Surveys focused on perceived usability, which assesses user satisfaction, typically involve recording subjective feelings experienced by individuals while using the system. This study is designed to thoroughly assess the usability of a CBCT viewer interface, focusing on its effectiveness, efficiency, and user satisfaction. It also aims to investigate the impact of demographic factors on usability outcomes and examine dental residents' experiences as primary users through the use of a predetermined set of questions. These facets must be addressed and resolved comprehensively to enhance user adoption of the system. Furthermore, usability issues can significantly hinder the adoption of information systems, leading to an increase in user-related mistakes and dissatisfaction [7]. Previous research has suggested that usability problems can contribute to medication errors, including serious mistakes when entering medication dosages into patients' healthcare records [7,8]. Similarly, a lack of proficiency in using the imaging software interface in dentistry may lead to delays in the diagnostic process and planning errors. Moreover, from a practical perspective, the inability to evaluate images may result in retakes or exploratory surgery, both of which could adversely affect the quality of care.

To the best of our knowledge, no previous study has investigated the usability aspects of a PACS processing CBCT data from the perspective of its active users. This study is designed to thoroughly assess the usability of a CBCT viewer interface, focusing on its effectiveness, efficiency, and user satisfaction. It also aims to investigate the impact of demographic factors on usability outcomes and to examine the dental professionals' experiences as primary users.

## Materials and methods

Ethical approval

This study followed the Declaration of Helsinki on medical protocol and ethics and all procedures followed were in accordance with the ethical standards of the responsible committee on human experimentation (institutional and national) and with the Helsinki Declaration of 1975, as revised in 2008. Informed consent was obtained from all participants to be included in the study. The Istanbul University Faculty of Dentistry Non-interventional Clinical Research Ethics Committee approved the study (Protocol no: 2023/20).

Sample size estimation

The sample size calculation was conducted using G*Power 3.1.9.7 software (Heinrich Heine University, Düsseldorf, Germany). The analysis protocol was based on including the minimum number of participants required to produce a statistically significant difference, with a predetermined cut-off score of 68 for the system usability scale (SUS-TR) total score variable. For this, we selected the "difference from constant" option from the t-test family (one sample case) and the "a priori" analysis option. Based on a pilot study with 10 participants, the mean SUS-TR total score variable was found to be 59.26 with a standard deviation (SD) of 12.88, resulting in an effect size of 0.67. Utilizing a two-tailed design, the same effect size, an error probability of 0.05, and a power of 0.80 (1-β), the non-centrality parameter was calculated to be 3.03, the critical t-value was 2.09, and the total sample size was determined to be 20. To account for non-parametric comparisons and potential dropouts, this number was increased by 10%, resulting in a final sample size of 22 participants (N=22).

Characteristics of the study participants

The inclusion criteria for the study were as follows: consenting to participate, working in the institution where the research project was conducted, having completed at least the first year of the residency program in oral and maxillofacial surgery, or having at most three months remaining in their official training period and, possessing the necessary language skills for the study's data collection tools.

CBCT data and PACS system

The CBCT dataset used in this study was obtained from a patient randomly selected among those who had visited the clinic in the past six months and had given consent to participate in the study. The patient's medical history and oral examination did not reveal any systemic or local conditions that could affect the radiographic appearance of the mandibular bone structure. Following image acquisition with Scanora 3Dx CBCT device (Dexis Inc., Quakertown, United States) using a 14x16 cm field of view, 0.2 mm^3^ isotropic voxel size, and 1 mm secondary reconstruction interval parameters, the tomographic data were uploaded to the PACS system. The acquired volume included the entire mandible, and no volume cutting was applied. Both mental foramen were inspected by an expert radiologist who confirmed that they exhibited standard anatomical characteristics in terms of number, shape, and position. All participants conducted their operations on this dataset for the remainder of the study.

Experimental settings and hardware

**Figure 1:**
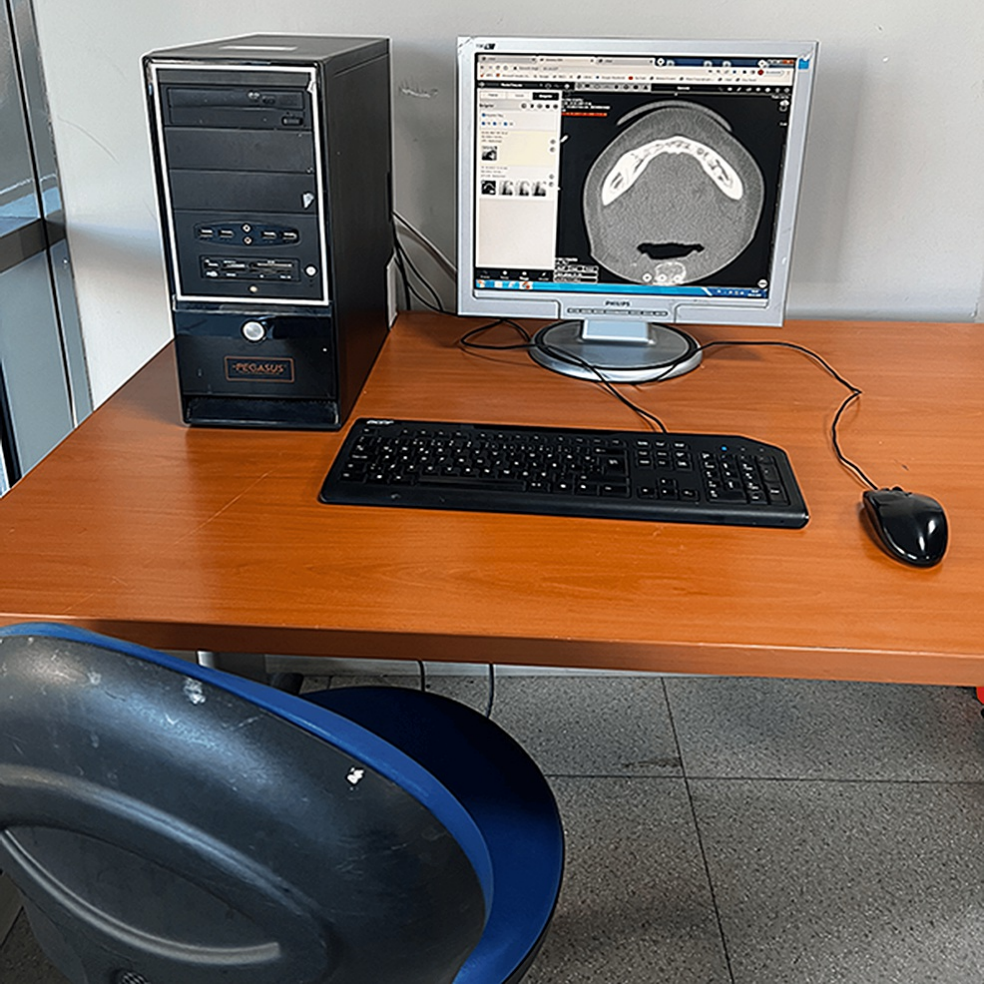
Representative photograph of the experimental settings and peripheral devices

The experimental process took place in a clinic room where the participants worked daily (Figure 1). For this experiment, a standard personal computer with an Intel I7 processor and 8 GB of installed memory (RAM) running the 64-bit Windows 7 operating system was used. The graphics card used in this system was from the Nvidia series Ge Force Gtx 730 with 4GB of RAM. This video card was connected to a 19-inch Philips liquid crystal display monitor, via a High-Definition Multimedia Interface cable. The screen resolution was set at 1280x1024 pixels, and a refresh rate of 60 Hz was applied. Peripheral devices connected to the system included an Acer KB-0759 keyboard and an A4Tech OP-620D mouse. The entire system was placed on a standard computer desk, with a standard computer chair positioned in front of it. Throughout the study period, no modifications were made to the computer system or its location.

Demographic data collection

At the beginning of the first session, the observer provided participants with a form containing demographic information. This form included details such as the participant's age, gender, years of experience as a resident, and self-reported proficiency level in using the Windows operating system (categorized as low, medium, or high). Additionally, the form included inquiries about their self-reported experience level with the PACS system (ranging from very low to very high) and their eyesight condition (classified as none, myopia, astigmatism, nearsightedness, farsightedness, or other). The collected data was then categorized into groups based on PACS and Windows experience, categorized as either below or above average, and eyesight was classified into categories such as no vision issues, myopia, both myopia and astigmatism. Years of residency were categorized as either below or above two years.

Task presentation

After completing the demographic form, the observer read the following text aloud to each participant: Thank you for participating in this study. This research is focused on evaluating a software interface and does not aim to test your professional skills or competence. Please start the PACS program on the computer desktop in front of you. Adjust the window settings in the MPR display mode to your preference. Use the pan and zoom functions and show the left or right mental foramen by rotating the axes in each of the axial, coronal, and sagittal plane windows. Please notify me when you are done.

Objective determinants of the usability

**Figure 2:**
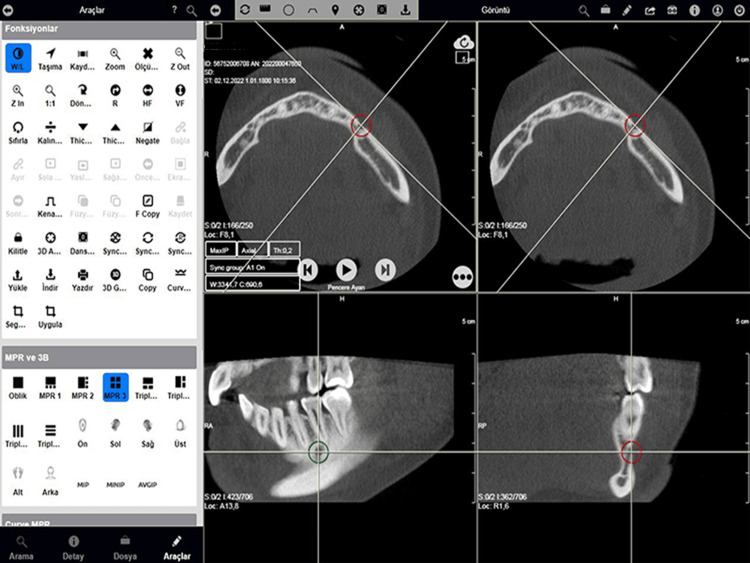
A screenshot taken at the end of a session during experiments showing the PACS interface The mental foramen from axial, coronal, and sagittal planes in curved multiplanar reconstruction display mode. PACS: Picture Archiving and Communication Systems

The observer waited for the participants to finish and recorded whether or not they completed the task as a dichotomous variable (effectiveness). No time restrictions were imposed (Figure 2). Throughout the sessions, without the participant's knowledge, task completion time, the number of mouse button clicks, and the distance of the mouse's on-screen path were recorded in both pixels and meters (efficiency).

Subjective determinants of usability

Upon completing the procedure, participants were provided with the SUS-TR and computer system usability questionnaire (T-CSUQ) questionnaires [9,10], which were translated versions of the originals [11,12] that had undergone validation, reliability testing, and factor analysis. The SUS-TR questionnaire comprises 10 questions in both positive and negative tones. In this study, SUS-TR scores were assessed on a 100-point scale without utilizing subscales. A higher score on the SUS-TR indicates better usability. The T-CSUQ scale consists of 13 questions, rated on a 100-point scale. It was evaluated based on both the total score and three subscale scores: system usefulness (SYSUSE), information quality (INFOQUAL), and interface quality (INTERQUAL). The scale is inverted, with a lower score indicating better usability. In the final phase of the experimental protocol, participants were asked to provide three comments about the system they had used in an open-ended format.

Interpretation of the usability scales

SUS-TR scores below 68 were categorized as below average, as suggested by Lewis and Sauro [13]. SUS-TR scores were also assessed using various scales, including the net promoter score (NPS) scale [14], adjective scale, acceptability scale, grade scale, and percentage rank [15,16]. The NPS classifies users into three categories based on their responses to a question rated from 0 to 10: promoters (scoring 9 and 10), passives (scoring 7 and 8), and detractors (scoring 6 and below). Bangor et al.'s [16] adjective scale uses descriptors like "best imaginable," "OK," and "worst imaginable" to evaluate the usability of the product. Another method for assessing the acceptability of SUS-TR involves using terms such as "acceptable" or "not acceptable" based on the average score. The range between 50 and 70 was defined as "marginally acceptable," which corresponds to a range from C to D on the grading scale. Grade scores are closely related to percentage rankings, which range from A to F.

Statistical analysis

The dataset was analyzed with IBM SPSS Statistics for Windows, Version 20 (Released 2011; IBM Corp., Armonk, New York, United States). The mean, median, and SDs of the study groups were used for descriptive statistics. The normality of the continuous variables was checked with the Shapiro-Wilk test and graphic methods. Independent samples t-test and analysis of variance (ANOVA) tests were used, respectively, for pairwise and multiple comparisons of the normally distributed data. Mann-Whitney U test was employed for non-parametric pairwise comparisons. The strength and significance of bivariate correlations were determined using the Pearson correlation coefficient. The confidence interval was set to 95% and p<0.05 was considered significant.

## Results

Based on the demographic information provided in the study, 27.3% of the 22 participants (N=22) were male, while 72.7% were female. The majority of the participants were second-year assistants (40.92%). Additionally, 54.5% of them reported having a good level of proficiency in using the Windows operating system. In terms of visual problems, 11 participants stated that they had no visual impairments, while the most common compensated visual defect was myopia (36.4%). All participants had prior experience with the PACS system, and a total of 81.8% reported using it frequently or always.

Since all participants successfully completed the given task, the task completion or effectiveness was rated as 100%. In terms of efficiency, the average time taken to complete the task was 177.18±92.55 seconds, with an average of 73.81±62.16 mouse clicks. The average mouse movement distance during the study was measured as 49.44±29.91 pixels and 17.53±10.65 meters in physical distance.

Regarding perceived usability or satisfaction, the study yielded an average SUS-TR total score of 60.68±19.58, with only 26.9% of participants’ scores falling within acceptable limits. The PACS system, as indicated by the SUS-TR total score, was classified as a "detractor" on the NPS scale, "marginal" on the acceptability scale, "OK" on the adjective scale, and "D" on the grade scale, with the average SUS-TR total score placing it at the 28th percentile. In terms of T-CSUQ test responses, the average total score was 43.63±16.34. Subscale means were 9.77±4.89 for INTERQUAL, 13.31±3.79 for INFOQUAL, and 16.68±7.91 for SYSUSE. Analysis of open-ended comments highlighted common negative issues, including difficulty accessing the MPR window, mouse cursor behavior (not changing shape to notify the user about its function), and non-descriptive error messages. Notably, no statistically significant difference emerged between the objective and subjective usability variables when reclassified and compared based on demographic parameters (Table 1).

**Table 1:** The effects of participants' demographic characteristics on the usability-related variables Means and standard deviations of effectiveness (task completion time), efficiency (number of mouse clicks, distance in pixel and meter), and satisfaction-related (SUS-TR total and T-CSUQ total) variables are stratified by participants’ demographic background. s: Seconds; SUS-TR: System usability scale; T-CSUQ: Computer system usability questionnaire

Variable	Sub-group	Task completion time	Number of mouse clicks	Distance in pixel	Distance in meter	SUS-TR total	T-CSUQ total
Gender	Male	203.5±22.2	89.5±25.89	57.86±13.44	20.57±4.87	50±19.81	54.50±15.04
	Female	167.31±25.76	67.93±15.68	46.29±7.29	16.39±2.57	64.68±18.52	39.56±15.28
Previous experience	Below average	185.25±29.93	71.75±33.44	47.25±14.54	16.90±5.51	57.50±10.60	49.25±7.13
	Above average	175.38±102.0	74.27±67.64	49.93±32.66	17.67±11.61	61.38±21.23	42.38±17.66
Windows experience	Below average	190.20±104.03	82.80±49.25	57.08±28.02	20.48±10.01	57.50±18.33	43.20±12.12
	Above average	166.33±84.95	66.33±72.52	43.08±31.12	15.08±10.97	63.33±20.97	44.00±19.73
Compensated vision status	None	167.27±93.63	74.81±75.13	49.22±34.06	17.46±12.07	58.40±24.45	44.63±19.73
	Myopia	184.00±110.52	62.62±32.56	45.67±21.90	16.25±7.89	64.37±16.35	41.62±15.36
	Myopia and astigmatism	195.33±46.19	100.00±84.32	60.33±41.01	21.21±14.81	59.16±3.81	45.33±2.51
Residency status	Less than two years	165.22±54.97	72.44±53.42	45.77±27.82	16.23±10.03	61.66±8.83	44.88±7.57
	More than two years	185.46±113.11	74.76±69.70	51.99±32.13	18.43±11.38	60±24.85	42.76±20.67

The average of the SUS-TR total and T-CSUQ total score exhibited a statistically significant negative correlation (r=-0.89, p<0.001). Additionally, the SUS-TR total score demonstrated a significant negative correlation with the number of clicks (r=-0.41, p=0.05), mouse distance in pixels (r=-0.42, p=0.04), mouse distance in meter (r=-0.43, p=0.04). and completion time (r=-0.45, p=0.03) (Tables 2, 3).

**Table 2:** Mean and standard deviation (SD) of the individual item scores of the system usability scale

Item number	Survey questions	Mean±SD score
1	I think that I would like to use this system frequently.	3.73±1.12
2	I found the system unnecessarily complex.	2.14±1.21
3	I thought the system was easy to use.	3.68±0.95
4	I think that I would need the support of a technical person to be able to use this system.	3.27±1.32
5	I found the various functions in this system were well integrated.	3.45±1.06
6	I thought there was too much inconsistency in this system.	2.05±1
7	I would imagine that most people would learn to use this system very quickly.	3.36±1.22
8	I found the system very cumbersome to use.	2±0.87
9	I felt very confident using the system.	2.77±1.07
10	I needed to learn a lot of things before I could get going with this system.	3.27±1.45
	Total score	60.68±19.58

**Table 3:** Mean and standard deviation (SD) of the individual item scores and subdomains of the computer system usability questionnaire

Item number	Survey questions	Mean±SD score
1	Overall, I am satisfied with how easy it is to use this system	2.73±1.64
2	It was simple to use this system	2.82±1.65
3	I am able to efficiently complete my work using this system	2.73±1.58
4	I feel comfortable using this system	2.91±1.6
5	It was easy to learn to use this system	2.95±1.4
6	I believe I became productive quickly using this system	2.55±1.41
7	The system gives error messages that clearly tell me how to fix problems	5.14±1.36
8	The information provided with this system is clear	4.68±1.49
9	The information provided for the system is easy to understand	3.5±1.5
10	The interface of this system is pleasant	3.36±2.04
11	I like using the interface of this system	3.41±1.97
12	This system has all the functions and capabilities I expect it to have	3±1.41
13	Overall, I am satisfied with this system	3.86±2.1
	Total score	43.64±16.35
Subdomains	System usefulness (items 1-6)	16.68±7.92
	Information quality (items 7-9)	13.32±3.8
	Interface quality (items 10-12)	9.77±4.9

## Discussion

This study investigated the usability of a PACS interface that processes CBCT data in a university hospital, with active residents acting as end-users. The usability of PACS interfaces plays a pivotal role in the efficiency and accuracy of diagnostic processes in medical imaging, directly impacting patient outcomes by facilitating quicker and more reliable interpretation of diagnostic images. Such high usability is crucial for reducing the cognitive load on healthcare professionals, thereby minimizing the risk of diagnostic errors and enhancing workflow efficiency within radiology departments. Furthermore, improving the usability of PACS interfaces is essential for their seamless integration into clinical workflows, ensuring that dental and medical professionals can access and interpret imaging data with greater ease and precision, thus significantly improving the quality of patient care. The inclusion of user-based evaluations in this process, a key aspect of the experimental approach, offers the advantage of obtaining real usage data, pinpointing significant issues, and conducting both formative and summative tests concurrently. The participants in this study shared a similar age range, educational background, and cultural-linguistic contexts. Despite the 23-year history of CBCT imaging, its inclusion in basic curricula occurred much later. Consequently, a considerable number of currently active dental professionals lack standard university-level training in this field. In addition to having this formal education, the participants' natural exposure to the rapid technological advances of the digital age during their formative years also influenced the selection of the sample. In dentistry, accessing the MPR Window, the norm leans towards a priori analyses and larger subject numbers for determining sample size; however, the field of human-computer interaction adopts a different stance. Nielsen [17] suggests that 75% of usability problems in a system can be identified with just five test subjects, while Macefield [18] suggested 8 to 25 users might provide a sensible range. To reconcile both perspectives, all eligible personnel were included in this study. They were also classified and analyzed based on their demographic characteristics.

When evaluating a software interface, factors like task complexity, user characteristics, and experimental settings can be expected to cause discrepancies. The mental foramen selected in this study is a relatively large structure and, therefore, it is clearly visible on most radiography techniques, minimizing the influence of radiological knowledge. This, in turn, provides more focus on testing the software interface rather than the participants’ individual competence. Instead of special laboratories, the present study opted for participants' everyday work environment and devices to ensure individual habits didn't affect examined variables. Objective variables of efficiency were favored for interface evaluation due to their frequent use and ease of implementation. The SUS-TR scale, the first survey after task completion for self-reported perceived usability, is widely used, unidimensional, and examined across different cultures [19]. In contrast, the T-CSUQ survey is multidimensional, delving into various usability facets [20]. The study results indicated a high correlation between the two surveys.

The definition of effectiveness within the usability concept is assessed based on the accuracy and completeness of users in achieving predetermined goals. Aldosari [21] investigated PACS acceptance in the radiology department using the technology acceptance model, concluding that there was no significant difference among participants. The study also noted that participants' age, gender, and PACS usage experience did not significantly impact acceptance levels. However, in a study by Bartella et al. [22], modeling impacted teeth, mandible fractures, and floor-of-mouth squamous cell carcinoma in an augmented/virtual reality (VR) environment using medical and cone beam CT data, it was observed that four users with varying professional experience level found it advantageous to easily examine the relationship between the distal and proximal segments in the mandibular fracture scenario. The authors also noted that as participants' professional experience increased, they derived less benefit from VR, and younger participants were more comfortable using VR glasses. In the present study, since all participants successfully completed the given task in the PACS system, the effectiveness is deemed to be 100%, albeit within the confines of this specific scenario. This finding can be considered predictable and, most likely, correlates with the task's simplicity and the majority of the participants being experienced with the interface. However, it's important to acknowledge that more complex tasks may yield different outcomes.

The efficiency of a system is typically assessed based on the speed of software responsiveness to user requests and how quickly users familiar with the interface design complete the assigned tasks. Accordingly, variables such as task completion time, the number of mouse or keyboard key presses, and cursor distance on the screen are commonly used in efficiency measurements. Willinger et al. [23] conducted a comparison of three different software applications used for planning the surgical correction of midface deformities. Participants were able to complete processes in all of them using the same datasets but significant differences were noted in terms of task duration. Piombino et al. [24] compared two software applications utilizing CBCT data for planning surgical treatment of dentofacial deformities in 10 patients. The authors considered variables such as task completion time, number of opened windows, and planning efficiency. Based on feedback from six surgeons, no significant difference was found between the two software applications. In the present study, no significant differences were observed with respect to professional experience level and system use experience among participants grouped according to demographic characteristics. This observation can be attributed to the participant's familiarity with the peripheral hardware and the interface itself. Additionally, a negative correlation was identified between task completion time, the number of mouse clicks and cursor distance on the screen, and user satisfaction. Consequently, it can be inferred that residents who complete tasks in the shortest and least movement-intensive manner may tend to report higher satisfaction in terms of the perceived usability of the interface.

Although the cut-off point for the SUS-TR score is 68, the average score of the system tested in the present study was 60.68. Additionally, according to the NPS, the PACS system in our study was deemed a detractor and garnered negative comments from users. Despite being 100% effective and with no individual variation in the demographic variables regarding efficiency, the SUS-TR scores being below average indicates issues with the software's interface. From this perspective, the subjective comments of the participants supported this observation. Challenges in using the software were reported to arise from the cursor on the interface not changing shape, unclear key meanings, and difficulty finding interface features. Sousa and Turrini [25], when testing a smartphone application for orthognathic surgery patients with 30 participants using the SUS-TR survey, reported an average total score of 79.84, with no correlation with patients' demographic characteristics. In another study, Zorzal et al. [26] stated that the average SUS-TR score of software designed for implant placement using CBCT data in a VR environment was 83.91. However, no evaluation was made based on the demographic characteristics of the participants. Gsaxner et al. [27] conducted usability tests of software showing the location of head and neck carcinoma lesions in real-time. It was determined that there was no difference in total system usage time with respect to previous experience and the system's average SUS-TR score was 74.8. Similarly, in their study examining the usability of virtual reality in digital surgical planning, Manzie et al. [28] found the average SUS-TR score of the system was 71.7. Despite being new designs, the SUS-TR scores were above average in these studies. Accordingly, the below-average SUS-TR score observed in the present one seems to be a rare occurrence. In line with the present study, Walji et al. [29] found out that the routinely used system's SUS-TR score was below average at 56.9. They also examined problems arising from the interface, workspace, and terminology, highlighting their negative impact on usability. Khundam et al. [30] similarly associated the SUS-TR scores with participants' negative comments. In accordance with prior empirical findings, it can be postulated that, notwithstanding prolonged utilization of the software system and the proficient execution of daily tasks by experienced operators, a substantial degree of dissatisfaction may persist in relation to its diverse functionalities.

Based on the open-ended feedback provided by the participants, many experienced difficulties in accessing the MPR window. To address this issue, we recommend implementing a more intuitive navigation system within the software, which could involve adding clearly labeled shortcuts and hover-over text descriptions to guide users more effectively. Furthermore, redesigning the interface layout to make the MPR window access point more prominent could significantly enhance accessibility. The feedback regarding cursor behavior underscores the need for a dynamic cursor that changes based on its current function or the button it is hovering over. We propose developing context-sensitive cursor icons that offer immediate visual feedback on the cursor's function, thereby reducing confusion and improving task efficiency. Additionally, the issue of non-descriptive error messages is critical in hindering effective troubleshooting by users. To overcome this, we suggest revising the error messaging system to include more informative and actionable messages.

This study had some limitations. The hardware specifications of the computer system and peripheral devices utilized in the experiments could have influenced the results. These details directly influence the speed and accuracy of image analysis as well as overall user satisfaction. To address the limitations identified in the study and enhance future research and clinical application, we recommend upgrading to more advanced hardware. This entails utilizing systems with greater processing power and memory, graphics units capable of efficiently handling high-resolution 3D imaging, and displays that offer clearer, more detailed visualizations. Additionally, selecting input devices designed for precision and ease of use can improve interaction with the software interface. Implementing these upgrades would likely overcome the challenges observed, leading to improvements in task efficiency, diagnostic accuracy, and user satisfaction, thus providing a more accurate assessment of CBCT viewer interfaces in demanding clinical settings. Moreover, as participants were presented with a single and straightforward scenario, drawing conclusions about other functionalities of the software based on the obtained data became challenging. Using diverse survey tools or advanced technology such as eye-tracking could yield more comprehensive insights into interface-related issues.

## Conclusions

The investigation of the usability of CBCT viewer interfaces among dental clinicians has illuminated several critical aspects of how they interact with advanced imaging technologies. Despite all participants completing the assigned task, the study revealed that the interface did not meet the expected usability standards, as evidenced by below-average scores. This outcome represents a fundamental disconnect between the design of dental imaging software and the practical needs and expectations of its users. The study's findings point to a pressing need for a user-centered design approach in the development of dental software which could be achieved by active engagement of end-users to the design and testing processes. In an era where digital technologies are becoming increasingly integral to healthcare delivery, the usability of such technologies can significantly influence clinical outcomes, patient safety, and professional satisfaction. Future research should focus on exploring innovative design solutions to address the usability challenges identified as well as extending the investigation to other dental software systems and technologies. By prioritizing usability, developers can enhance the adoption and effectiveness of digital technologies in dental practice, ultimately improving patient care and professional satisfaction.
